# Anti-Obesity and Hepatoprotective Effects of Herring–Saury Oil Fermented by *Lactobacillus brevis* KCCM13538P in High-Fat-Diet-Induced Mice

**DOI:** 10.3390/foods14162862

**Published:** 2025-08-18

**Authors:** Hyun-Sol Jo, Tae-Won Goo, Sun-Mee Hong

**Affiliations:** 1 Marin Industry Research Institute for East Sea Rim, 22 Haeyanggwahak-gil, Uljin-gun 36315, Gyeongsangbuk-do, Republic of Korea; hyunsoljo@kfri.re.kr; 2 Korea Food Research Institute, Iseo-myeon, Wanju-Gun 55365, Jeollabuk-do, Republic of Korea; 3 Department of Biochemistry, College of Medicine, Dongguk University, Gyeongju 38766, Gyeongsangbuk-do, Republic of Korea

**Keywords:** fermented fish oil, *Lactobacillus brevis*, high-fat diet, obesity, liver function

## Abstract

**Background:** Obesity-associated liver dysfunction is a key feature of metabolic syndrome. Marine by-products, such as fish oils, offer promising dietary interventions. In this study, we aimed to assess the anti-obesity and hepatoprotective effects of herring–saury by-product-derived fermented fish oil—Gwamegi oil (GmO)—and the same oil fermented with *Lactobacillus brevis* KCCM13538P (GmOLb) in a high-fat-diet (HFD)-induced obese mouse model. **Methods**: GmO was extracted and fermented. Anti-obesity and hepatoprotective effects were assessed using in vitro and in vivo studies. For the in vivo study, female C57BL/6J mice were fed an HFD supplemented with lard (control), GmO, or GmOLb for 60 days. Metabolic and liver function parameters were assessed. **Results**: In 3T3-L1 adipocytes, GmOLb significantly reduced lipid accumulation and intracellular triglyceride (TG) levels compared with GmO. In HFD-fed mice, GmOLb significantly reduced body weight gain, ovarian fat mass, serum TG, low-density lipoprotein cholesterol, leptin concentration, atherogenic indices, and cardiac risk factor ratio. Furthermore, it reduced liver damage indicators, including alanine aminotransferase, aspartate aminotransferase, and total bilirubin levels. **Conclusions**: Fermenting herring–saury oil with *L. brevis* KCCM13538P enhanced its anti-obesity and hepatoprotective effects in HFD-fed mice. GmOLb shows strong potential as a functional dietary lipid for preventing and managing metabolic disorders.

## 1. Introduction

Obesity is a major global health concern that significantly increases the risk of metabolic complications, including type 2 diabetes, non-alcoholic fatty liver disease (NAFLD), cardiovascular disease, and certain cancers. It results from a complex interplay of factors, including a sustained energy imbalance, reduced physical activity, and dysregulated lipid and glucose metabolism. The widespread adoption of Western-style diets, characterized by an excessive intake of saturated fats, refined carbohydrates, and ultra-processed foods, has further exacerbated the global prevalence of obesity and its associated complications [1,2]. High-fat diets (HFDs) are widely used in experimental models to replicate the key features of human obesity, such as visceral adiposity, hepatic steatosis, dyslipidemia, and chronic low-grade inflammation [3,4,5].

Marine-derived oils, particularly those rich in omega-3 polyunsaturated fatty acids (ω-3 PUFAs), have gained increasing attention for their anti-obesity and hepatoprotective properties [6,7]. Herring (*Clupea pallasii*) and saury (*Cololabis saira*) are two underutilized fish species rich in bioactive lipids, particularly eicosapentaenoic acid (EPA) and docosahexaenoic acid (DHA). These long-chain ω-3 PUFAs support lipid metabolism, reduce systemic inflammation, and enhance hepatic function [8,9,10]. In addition to ω-3 PUFAs, marine oils from herring and saury contain physiologically relevant levels of fat-soluble vitamins, including vitamins A, D, E, and K, which may aid immune regulation, lipid metabolism, and vascular health [11,12,13]. Among these, vitamin K2 variants such as menaquinone-4 (MK-4) and menaquinone-7 (MK-7) are notable for their high bioavailability and metabolic functions [14,15,16]. Gwamegi is a traditional Korean seafood product made by semi-drying herring or saury using cold sea winds during winter months. This artisanal preservation method, primarily practiced in coastal areas such as Pohang, enhances flavor and nutritional value while maintaining a soft, chewy texture. Gwamegi is widely consumed in Korea; however, it is relatively unfamiliar in global markets and scientific literature. In this study, oil extracted from surplus herring and saury raw materials—before their use in traditional Gwamegi production—is referred to as Gwamegi oil (GmO).

The functional efficacy of marine oils can be further enhanced through microbial fermentation using probiotic strains, such as *Lactobacillus brevis* [17,18]. Fermentation enhances the oxidative stability and bioavailability of unsaturated fatty acids (USFAs) and generates secondary metabolites with anti-inflammatory and lipid-lowering activities [19,20]. *L. brevis*, a lactic acid bacterium commonly identified in fermented foods, has demonstrated beneficial properties, including antioxidant, anti-obesity, and hepatoprotective effects [21]. Specifically, *L. brevis* possesses esterase and lipase genes that are enzymatically active under fermentation conditions, enabling the hydrolysis of triglycerides and esterified lipids [22]. In this study, we developed a probiotic fermentation system using a blend of herring and saury by-product oils inoculated with *L. brevis* KCCM13538P, supplemented with 0.1% yeast extract as a minimal nitrogen source to maintain microbial viability. In this study, we aimed to evaluate the anti-obesity and hepatoprotective effects of Gwamegi oil (GmO) and its fermented form (GmOLb) in in vitro and in vivo models of metabolic dysfunction. We hypothesized that fermentation with *L. brevis* KCCM13538P would enhance the functional efficacy of fish oil by increasing ω-3 PUFA bioavailability and generating additional bioactive metabolites [23,24]. To test this hypothesis, we assessed lipid accumulation, serum lipid profiles, liver function markers, and histopathological changes in HFD-induced obese mice.

## 2. Materials and Methods

### 2.1. Preparation of GmO and GmOLb

GmO was prepared by mixing the by-products of herring (*C. pallasii*) and saury (*C. saira*) at a 1:9 ratio, reflecting the relative availability of raw materials during production, as saury by-products were more abundant. Future studies will compare various blending ratios to evaluate their effects on the physicochemical properties and biological activities of the resulting oils. The raw materials, sourced from Hyun-Dae Feed Co., Ltd. (Pohang, Gyeongsangbuk-do, Republic of Korea), were thermally treated at 95 °C for 5 min to separate lipids from solid residues. Subsequently, the mixture was centrifuged (3000× *g*, 10 min) to obtain the oil fraction. The collected oil (GmO) was filtered using a separatory funnel to remove any insoluble particles, and stored at 4 °C for short-term use or at −20 °C for long-term storage. Prior to the experiments, GmO was equilibrated at room temperature for 1 h to ensure complete liquefaction. For fermentation, GmO supplemented with 0.1% yeast extract (BD Difco™, Sparks, MD, USA) was sterilized at 121 °C for 15 min to eliminate potential microbial contaminants. *L. brevis* KCCM13538P was used as the fermenting strain. A preculture was prepared by inoculating 1% (*v*/*v*) *L. brevis* KCCM13538P into de Man, Rogosa, and Sharpe broth (BD Difco™, Sparks, MD, USA), and incubating at 30 °C for 24 h. The resulting bacterial suspension was adjusted to an optical density of 1.0 (10^8^ CFU/mL) at 600 nm (OD_600_), and 1.0% (*v*/*v*) of this suspension was inoculated into the sterilized GmO. Fermentation was conducted under stationary conditions at 30 °C for 24 h, achieving a viable cell count of approximately 10^10^ to 10^11^ CFU/mL. The fermented oil, designated as GmOLb, was subsequently stored at −20 °C.

### 2.2. Fatty Acid and Fat-Soluble Vitamin Analysis

Fatty acid composition was determined using gas chromatography (GC) after derivatization to fatty acid methyl esters (FAMEs; Sigma-Aldrich, St. Louis, MO, USA) following the method described by Ichihara et al. [25], with minor modifications. Lipids were methylated with 14% boron trifluoride (Sigma-Aldrich, St. Louis, MO, USA) in methanol (Sigma-Aldrich, St. Louis, MO, USA), followed by extraction with hexane (Sigma-Aldrich, MO, USA). The resulting FAMEs were analyzed using a GC system (Agilent 7890A; Agilent Technologies, Santa Clara, CA, USA) equipped with a flame ionization detector and capillary column (HP-88; 100 m × 0.25 mm, 0.20 μm film thickness). The injector and detector temperatures were set at 250 °C, and the oven temperature was programmed to increase from 140 °C (held for 5 min) to 240 °C at a rate of 4 °C/min. Helium was used as the carrier gas at a flow rate of 1.0 mL/min. Individual fatty acids were identified by comparing retention times with those of authentic standards. Quantitative data were expressed as the mean ± standard deviation (SD), excluding trace or unidentified peaks.

Fat-soluble vitamins (A, D, E, and K2) were extracted using a liquid–liquid extraction method and analyzed by high-performance liquid chromatography (HPLC), following the previously described methods [26,27], with slight modifications. Oil samples were saponified with ethanolic potassium hydroxide and extracted using n-hexane. The extracted vitamins were analyzed using an HPLC system (Waters Alliance e2695; Waters Corp., Milford, MA, USA) equipped with a photodiode array detector. Separation was performed on a C18 reverse-phase column (250 × 4.6 mm, 5 μm). The mobile phase consisted of methanol and water (95:5, *v*/*v*) at a flow rate of 1.0 mL/min. Detection wavelengths were 325, 265, 290, and 248 nm for vitamins A, D, E, and K2, respectively. Vitamin A was expressed as retinol equivalents (RE), vitamin D and K2 (MK-4 and MK-7) as micrograms (μg), and vitamin E as alpha-tocopherol equivalents (α-TE). The vitamin content was normalized to 100 g of oil.

### 2.3. T3-L1 Cell Culture and Adipogenic Differentiation

Murine 3T3-L1 preadipocytes (American Type Culture Collection CL-173; Manassas, VA, USA) were cultured in Dulbecco’s modified Eagle’s medium (DMEM; Gibco, Grand Island, NY, USA) supplemented with 10% (*v*/*v*) bovine calf serum (HyClone, Logan, UT, USA) and 1% (*v*/*v*) penicillin–streptomycin (Gibco, Grand Island, NY, USA) at 37 °C in a humidified atmosphere containing 5% CO_2_. Upon reaching confluence (designated as day 0), adipogenic differentiation was induced by treating the cells for 48 h with a standard MDI induction cocktail containing 0.5 mM 3-isobutyl-1-methylxanthine (Sigma-Aldrich, St. Louis, MO, USA), 1 μM dexamethasone (Sigma-Aldrich, St. Louis, MO, USA), and 10 μg/mL insulin (Sigma-Aldrich, St. Louis, MO, USA) in DMEM supplemented with 10% (*v*/*v*) fetal bovine serum (FBS; Gibco, Grand Island, NY, USA), as described by Zebisch et al. [28], with minor modifications. As insulin was already included in the MDI cocktail to induce adipogenesis, it was not used separately as a positive control during the differentiation assay. After 48 h (day 2), the medium was replaced with DMEM containing 10% (*v*/*v*) FBS and 10 μg/mL insulin, and the cells were further incubated for six days with media changes every two days. Test samples, including GmO and GmOLb (fermented with *L. brevis* KCCM13538P), were administered at concentrations ranging from 10 to 300 μg/mL throughout the differentiation period (day 0 to day 8). Control cells were treated with vehicle dimethyl sulfoxide (DMSO) under similar differentiation conditions. Adipocyte differentiation and lipid accumulation were assessed on day 8, as described by Gregoire et al. [29].

### 2.4. Cell Viability and Lipid Accumulation Assays

Cell viability was assessed using the 3-(4,5-dimethylthiazol-2-yl)-2,5-diphenyltetrazolium bromide (MTT) assay (Abcam, Cambridge, UK). Then, 3T3-L1 preadipocytes were seeded in 96-well plates and treated with various concentrations (10–300 μg/mL) of GmO or GmOLb for 24 h. Subsequently, 20 μL of MTT solution (5 mg/mL in phosphate-buffered saline [PBS]; Sigma-Aldrich) was added to each well and incubated at 37 °C for 4 h. The resulting formazan crystals were solubilized in 150 μL of DMSO, and absorbance was measured at 570 nm using a microplate reader (BioTek Instruments, Winooski, VT, USA), as described previously [30]. Lipid accumulation was assessed using Oil Red O staining (Sigma-Aldrich) on day 8 of differentiation. Cells were washed with PBS, fixed with 10% (*v*/*v*) formalin for 30 min, and stained for 1 h at room temperature using a filtered Oil Red O solution (0.5% in isopropanol, diluted 3:2 [*v*/*v*] with distilled water). After thorough washing with distilled water, the stained intracellular lipids were visualized. For quantification, the retained dye was eluted with 100% isopropanol, and absorbance was measured at 520 nm using a microplate reader, as described by Ramirez-Zacarias et al. [31]. Intracellular triglyceride (TG) content was determined using a commercial Triglyceride Quantification Assay Kit (Abcam), following the manufacturer’s protocol. Absorbance was recorded at 570 nm and normalized to the total protein content that was measured using the bicinchoninic acid assay (Thermo Fisher Scientific, Waltham, MA, USA), following the method described by Smith et al. [32].

### 2.5. Animal Study Design

Thirty-two female C57BL/6 mice (5-weeks-old) were obtained from Daehan Bio Link Co., Ltd. (Eumseong, Republic of Korea) and housed under standard laboratory conditions (22 ± 2 °C, 55 ± 10% humidity, and 12 h light/dark cycle) with ad libitum access to food and water. After one week of acclimatization, the mice were randomly divided into four groups (*n* = 8 per group) as follows: ND: normal diet (AIN-76A), HFD_L: HFD containing 45% lard (AIN-76A modified), HFD_GmO: HFD containing 45% GmO, and HFD_GmOLb: HFD containing45 % GmOLb (fermented with *L*. *brevis* KCCM13538P). All diets were prepared based on the AIN-76A formulation (DooYeol Biotech, Seoul, Republic of Korea) and provided ad libitum for 60 days, adhering to the American Institute of Nutrition guidelines [33]. The HFD was formulated to provide 45% of total energy from fat. In the GmO and GmOLb groups, 45% (*w*/*w*) of the total dietary fat was replaced with either non-fermented or fermented GmO, replacing an equivalent amount of lard. Body weight and food intake were monitored weekly. At the end of the experiment, mice were fasted overnight, anesthetized with isoflurane (Sigma-Aldrich, St. Louis, MO, USA), and euthanized by cardiac exsanguination. Blood samples were collected via cardiac puncture, and the serum was separated by centrifugation (3000 rpm, 15 min, 4 °C). Liver, adipose, and other relevant tissues were excised, weighed, and either snap-frozen in liquid nitrogen or fixed in 10% (*v*/*v*) neutral-buffered formalin for histological and molecular analyses. No explicit inclusion or exclusion criteria were set a priori. All animals completed the study and were included in the analysis, as no signs of illness unrelated to the intervention were observed. The sample size (*n* = 8 per group) was determined based on conventional standards in similar preclinical models; no formal power calculation was performed. Animals were randomly assigned to groups; however, no specific measures were taken to control for potential confounders such as cage position or treatment order. Group allocation and outcome assessments were not blinded. We acknowledge that the absence of blinding and a formal power calculation is a limitation of this study, and we will incorporate these elements in future experimental designs to enhance rigor and reproducibility. All animal procedures were approved by the Institutional Animal Care and Use Committee (IACUC) of Dongguk University (approval no. IACUC-2024-04; approval date: 25 April 2024).

### 2.6. Serum Biochemical Analysis

At the end of the experimental period, blood was collected via cardiac puncture under anesthesia and allowed to clot at room temperature for 30 min. Serum was separated by centrifugation (3000 rpm, 15 min, 4 °C) and stored at −80 °C. Serum levels of TG, total cholesterol (TC), high-density lipoprotein cholesterol (HDL-C), low-density lipoprotein cholesterol (LDL-C), alanine aminotransferase (ALT), and aspartate aminotransferase (AST) were measured using commercial enzymatic colorimetric assay kits (Asan Pharmaceutical Co., Ltd., Seoul, Republic of Korea), following the manufacturer’s protocol. Serum samples were mixed with specific enzyme reagents in 96-well microplates, and the resulting colorimetric reactions were quantified using a microplate reader (BioTek Instruments, Winooski, VT, USA) at the appropriate wavelengths (550 nm for TG and 500 nm for cholesterol). Serum leptin concentrations were determined using mouse-specific enzyme-linked immunosorbent assay kits (R&D Systems, Minneapolis, MN, USA). Assays were performed following the manufacturer’s protocol. OD was measured at 450 nm, and the concentrations were determined using standard curves prepared with known concentrations of recombinant mouse leptin. All measurements were performed in triplicate and are expressed as the mean ± SD. Inter- and intra-assay variability was maintained within 10%.

### 2.7. Statistical Analysis

Statistical analyses were performed using SPSS software (version 20.0; SPSS Inc., Chicago, IL, USA). Data are presented as the mean ± standard deviation (SD). Normality was assessed using the Shapiro–Wilk test. A one-way analysis of variance (ANOVA), followed by Tukey’s honest significant difference (HSD) test, was used to determine statistical significance, which was set at *p* < 0.05. The sample size (*n* = 8 per group) was based on precedent in similar preclinical studies; no formal power analysis was conducted.

## 3. Results

### 3.1. Fatty Acid Composition of GmO and GmOLb

The fatty acid compositions of GmO and GmOLb are presented in Table 1. Fermen-tation with *L*. *brevis* KCCM13538P resulted in a modest reduction in total saturated fatty acids (SFAs) and a corresponding increase in USFAs, particularly ω-3 PUFAs. Notably, the levels of EPA (C20:5 ω-3) and DHA (C22:6 ω-3) were slightly elevated in GmOLb than in GmO. However, independent t-test analysis revealed that these differences were not statistically significant (*p* > 0.05). Fatty acid data were analyzed using independent t-tests to compare the means between the GmO and GmOLb groups. Statistical analyses were conducted using SPSS software (version 20.0; SPSS Inc., Chicago, IL, USA), and results are expressed as mean ± SD (*n* = 3). These long-chain ω-3 PUFAs are well known for their anti-inflammatory, lipid-lowering, and cardiometabolic benefits [34,35,36,37]. Thus, the compositional changes suggest a potential fermentation-induced enhancement; however, further studies with larger sample sizes and targeted enzymatic assays are needed to confirm the functional relevance.

While previous research has reported lipase and esterase activities in *L. brevis* [22], its substrate specificity toward esterified ω-3 PUFAs remains unclear. Since enzymatic activity was not directly quantified in this study, the observed increase in ω-3 content may instead reflect enhanced oxidative stability or bioavailability post-fermentation. Future investigations using both targeted enzymatic assays and untargeted metabolomics are needed to clarify the underlying biochemical mechanisms.

### 3.2. Contents of Fat-Soluble Vitamins

Fat-soluble vitamins (A, D, E, and K2) play crucial roles in antioxidant defense, bone metabolism, and overall metabolic regulation [38,39,40]. Because of the lipophilic nature of marine oils, fermentation may affect the stability, transformation, or biosynthesis of these vitamins through microbial enzymatic pathways, such as esterification, dehydrogenation, or decarboxylation [41,42]. In this study, we assessed the effect of *L. brevis* KCCM13538P fermentation on the fat-soluble vitamin content of GmO to assess the potential for nutritional enhancement through microbial bioprocessing. The concentrations of fat-soluble vitamins in non-fermented GmO and GmOLb are summarized in Table 2. Fermentation significantly altered the vitamin profile, specifically enhancing the bioactive compound levels. The vitamin A content, expressed as RE, increased from 6999.41 ± 34.99 μg/100 g in GmO to 7579.27 ± 37.89 μg/100 g in GmOLb. This may be attributed to the microbial-mediated conversion of provitamin A carotenoids (β-carotene) to retinoid forms or the increased stability of pre-existing retinoids during fermentation [43]. Furthermore, the vitamin D content increased significantly, from 56.91 ± 0.28 μg/100 g in GmO to 67.95 ± 0.34 μg/100 g in GmOLb. This result is nutritionally significant because of the crucial role of vitamin D in calcium homeostasis, immune regulation, and metabolic health [44]. Lactic acid bacteria affect vitamin D metabolism and protect against its degradation in lipid-rich matrices [45]. A significant increase in vitamin E (α-TE) was observed, from 1070 ± 6.85 μg to 4780 ± 23.91 μg/100 g, after fermentation. Due to its role as a major lipid-phase antioxidant, this increased tocopherol level may contribute to enhanced oxidative stability and bioactivity of the fermented oil [46], possibly enhancing its shelf-life and physiological functions. For vitamin K2, both MK-4 (from 42.19 ± 3.26 to 64.13 ± 3.07 μg) and MK-7 (from 19.28 ± 1.48 to 46.56 ± 1.82 μg/100 g) contents were significantly increased in GmOLb. These forms of vitamin K2 are associated with bone health, vascular calcification prevention, and anti-inflammatory effects [47,48]. The observed enrichment indicates that *L. brevis* may harbor metabolic pathways for MK biosynthesis or facilitate conversion from dietary phylloquinone during fermentation [49]. In summary, these results indicate that microbial fermentation of GmO preserves and significantly enhances its nutritional profile, specifically in terms of bioactive fat-soluble vitamins. The increased levels of vitamins A, D, E, and K2 may synergistically contribute to the observed anti-obesity and hepatoprotective effects of GmOLb, potentially through antioxidant activity, lipid metabolism regulation, and systemic metabolic support.

### 3.3. Effects on Lipid Accumulation in 3T3-L1 Adipocytes

**Figure 1:**
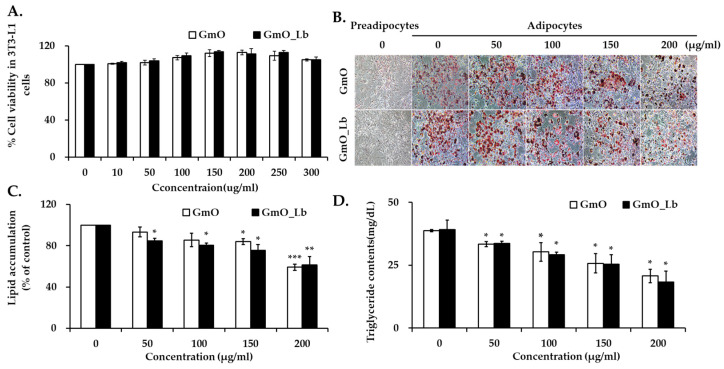
Effects of GmO and GmOLb on lipid accumulation in 3T3-L1 adipocytes. (**A**) Cell viability of 3T3-L1 preadipocytes treated with GmO or GmOLb at concentrations ranging from 10 to 300 µg/mL for 24 h, assessed by the MTT assay. (**B**) Representative images of Oil Red O staining to visualize intracellular lipid accumulation during adipocyte differentiation in response to GmO or GmO_Lb treatment. (**C**) Quantitative analysis of intracellular lipid content based on the absorbance measurements of eluted Oil Red O dye from stained adipocytes. (**D**) Intracellular TG content in differentiated 3T3-L1 adipocytes treated with GmO or GmO_Lb. All data are presented as the mean ± standard deviation (*n* = 3). Statistical significance is indicated as * *p* < 0.05, ** *p* < 0.01, and *** *p* < 0.001 vs. control group. GmO, Gwamegi oil; GmOLb, Gwamegi oil fermented with *L. brevis* KCCM 13538P; TG, triglyceride; MTT, 3-(4,5-dimethylthiazol-2-yl)-2,5-diphenyltetrazolium bromide.

To assess the anti-obesity potential of GmO and GmOLb, their effects on lipid accumulation were assessed during 3T3-L1 adipocyte differentiation. As illustrated in Figure 1A, treatment with GmO and GmOLb at concentrations up to 300 µg/mL did not exhibit significant cytotoxicity in 3T3-L1 preadipocytes, as determined by the MTT assay. These results confirm the biocompatibility of both oils and support their suitability for further adipogenic studies. Oil Red O staining (Figure 1B) revealed significant intracellular lipid accumulation in the control group. In contrast, both GmO- and GmOLb-treated groups exhibited visibly reduced lipid droplet formation. Quantitative analysis of Oil Red O absorbance (Figure 1C) demonstrated a dose-dependent inhibition of lipid accumulation by GmO and GmOLb. Although GmO exhibited slightly lower lipid accumulation than that of GmOLb at 200 μg/mL, GmOLb exerted superior anti-lipogenic effects at most concentrations. Notably, at 200 μg/mL, GmOLb reduced lipid content by approximately 45% compared to that of the control group (*p* < 0.01), indicating enhanced efficacy through fermentation. Additionally, intracellular TG levels were significantly reduced in both GmO- and GmOLb-treated adipocytes (Figure 1D), further supporting their lipid-lowering capacity. GmOLb exhibited significant TG-lowering effects than that of GmO at all tested concentrations except 50 μg/mL, indicating enhanced bioactivity through fermentation. The superior anti-adipogenic effect of GmOLb may be attributed to its high levels of ω-3 PUFAs (EPA and DHA) and fat-soluble vitamins (A, D, E, and K2) (Table 1 and Table 2). These components regulate adipogenesis and lipid metabolism by modulating peroxisome proliferator-activated receptor gamma (PPARγ) signaling, reducing oxidative stress, and enhancing mitochondrial function [50,51,52]. In summary, these findings indicate that lactic acid bacterial fermentation of marine oils, such as GmO, may enhance their efficacy in reducing adipocyte lipid accumulation. GmOLb is a promising candidate as a functional food or nutraceutical for targeting obesity and related metabolic disorders.

### 3.4. Effects on Body Weight Gain and Adiposity in HFD-Fed Mice

**Figure 2:**
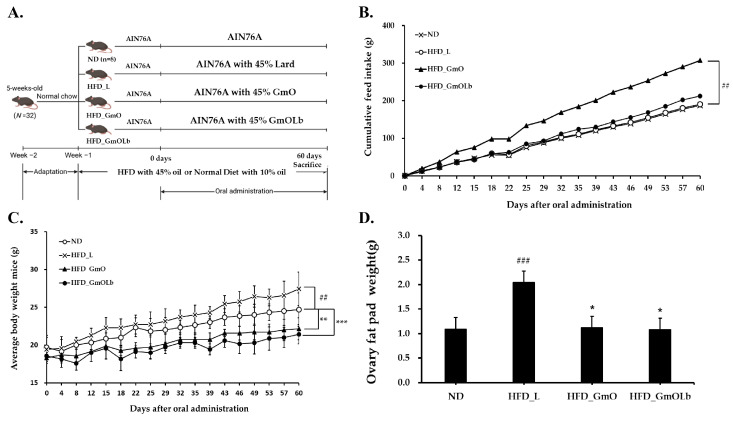
Effects of GmO and GmOLb on body weight gain and adipose tissue accumulation in HFD-fed mice. (**A**) Experimental design. Five-week-old female mice (*n* = 32) were assigned to four groups (*n* = 8 each): ND (normal diet), HFD_L (HFD with 45% lard), HFD_GmO (HFD with 45% Gwamegi oil), and HFD_GmOLb (HFD with 45% *L. brevis*-fermented Gwamegi oil). After a 1-week acclimation, diets were administered for 60 days. (**B**) Cumulative feed intake during the 60-day period. (**C**) Body weight alterations during the feeding period. (**D**) Ovary fat pad weight at sacrifice. Data are presented as the mean ± standard deviation (*n* = 8). Statistical significance: ^##^ *p* < 0.01 and ^###^ *p* < 0.001 vs. ND; * *p* < 0.05, ** *p* < 0.01, and *** *p* < 0.001 vs. HFD_L. ND, normal diet; HFD, high-fat diet; GmO, Gwamegi oil; GmOLb, Gwamegi oil fermented with *L. brevis* KCCM 13538P.

To assess the anti-obesity effects of GmO and GmOLb, we measured body weight gain, cumulative feed intake, and visceral adiposity in an HFD-induced obesity mouse model (Figure 2A). Gm-O exhibited the highest feed intake among the HFD-fed groups; however, differences in body weight were observed between the treatment groups, indicating that variations in weight gain were not attributable to differences in feed consumption (Figure 2B). As anticipated, the HFD_L group exhibited significantly higher body weight gain than that of the ND group (Figure 2C). Both GmO- and GmOLb-supplemented HFD attenuated weight gain, with GmOLb producing a more significant suppression. Ovary fat pad mass was significantly increased in the HFD_L group than that in the ND group (*p* < 0.001). However, both the HFD_GmO and HFD_GmOLb groups exhibited significant reductions (*p* < 0.05), with no difference between them (Figure 2D). These findings indicate that GmO alleviates HFD-induced visceral fat accumulation and that fermentation with *L. brevis* augments the anti-obesity efficacy of the extract.

Fatty acid composition analysis revealed that both GmO and GmOLb contained significantly higher levels of PUFAs, specifically EPA and DHA (Table 1). Following fermentation with *L. brevis*, GmOLb exhibited a slight increase in overall ω-3 PUFAs, whereas ω-6 PUFA levels also increased modestly, resulting in only a minor enhancement in the ω-3/ω-6 PUFA ratio (from approximately 5.10 to 5.05). Despite this subtle shift, the increased absolute concentrations of ω-3 PUFAs—recognized for their anti-adipogenic and lipid-oxidizing effects—contributed to the favorable lipid profile of GmOLb. Notably, fermentation significantly reduced the SFA content in GmOLb. The co-occurrence of high PUFA and reduced SFA levels enhances mitochondrial β-oxidation and suppresses adipogenesis, processes associated with reduced adipose tissue mass [53,54]. These compositional alterations likely highlight the reduced fat accumulation observed in the GmOLb-treated group. In addition, fermentation was associated with increased levels of bioactive lipid components, including medium-chain triglycerides (MCTs) and conjugated linoleic acid (CLA). MCTs rapidly undergo hepatic β-oxidation and facilitate energy expenditure through thermogenesis [55], whereas CLA modulates lipid metabolism by activating PPARγ and uncoupling protein 1 in the adipose tissue [56,57]. Moreover, GmOLb exhibited increased levels of fat-soluble vitamins, including MK-4, MK-7, A, D, and E (Table 2). These micronutrients play crucial roles in enhancing insulin sensitivity, antioxidant defense, and metabolic regulation [58,59,60]. Specifically, MK-4 enhances insulin secretion and supports glucose homeostasis in both preclinical and clinical studies [61].

In summary, these findings indicate that GmOLb exhibits superior anti-obesity effects than that of unfermented GmO, likely because of compositional enhancements achieved through microbial fermentation. The altered fatty acid profile, increased levels of MCTs and CLA, and enrichment of functional lipid micronutrients highlight the potential of lactic acid bacteria-mediated fermentation as an effective strategy for enhancing the metabolic benefits of marine-derived oils.

### 3.5. Effects on Serum Lipid Profiles and Cardiovascular Risk in HFD-Fed Mice

**Figure 3:**
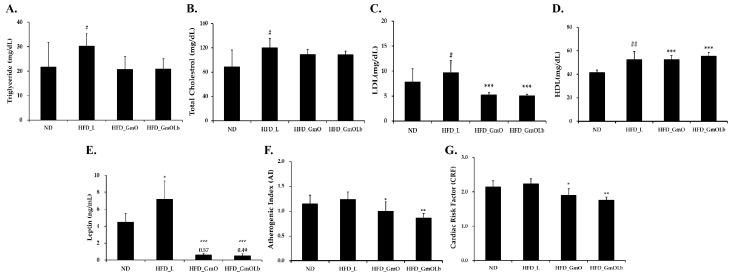
Effects of GmO and GmOLb on serum lipid profiles and cardiovascular risk markers in HFD-fed mice. (**A**–**D**) Serum levels of TG, TC, LDL-C, and HDL-C after 60 days of dietary intervention. (**E**–**G**) Leptin, AI, and CRF values in each treatment group. Data are presented as the mean ± standard deviation (*n* = 8 per group). Statistical significance: ^#^ *p* < 0.05 and ^##^ *p* < 0.01 vs. ND; * *p* < 0.05, ** *p* < 0.01, and *** *p* < 0.001 vs. HFD_L. ND, normal diet; HFD, high-fat diet; GmO, Gwamegi oil; GmOLb, Gwamegi oil fermented with *L. brevis* KCCM13538P; TG, triglycerides; TC, total cholesterol; LDL-C, low-density lipoprotein cholesterol; HDL-C, high-density lipoprotein cholesterol; AI, atherogenic index; CRF, cardiac risk factor. AI = (TC–HDL-C)/HDL-C; CRF = TC/HDL-C.

After a 60-day intervention in HFD-fed mice, we measured serum lipid and cardiometabolic marker levels to assess the effects of GmO and GmOLb (Figure 3). As anticipated, HFD_L mice exhibited significant hyperlipidemia—TG, TC, and LDL-C, alongside increased HDL-C—confirming diet-induced dyslipidemia (Figure 3A–D). Supplementation with either GmO or GmOLb significantly lowered TG and TC levels while restoring HDL-C levels compared to HFD_L. Both the GmO and GmOLb groups exhibited a significant effect, with TG and LDL-C levels reduced to nearly those of the ND group, and HDL-C levels significantly increased. This indicates that GmO has a lipid-modulating efficacy. Serum leptin, an adipose-derived hormone implicated in energy regulation and leptin resistance, increased from approximately 4 ng/mL in ND mice to 7 ng/mL in HFD_L. Both GmO and GmOLb significantly reduced leptin levels (to approximately 0.57 and 0.49 ng/mL, respectively; *** *p* < 0.001 vs. HFD_L) (Figure 3E), indicating enhanced leptin sensitivity and adiposity reduction. These leptin-lowering effects align with previous studies demonstrating that vitamin K2 supplementation reduces circulating leptin levels in obese animal and clinical models [62].

To quantify cardiovascular risk, we calculated the atherogenic index (AI = [TC–HDL-C]/HDL-C) and cardiac risk factor (CRF = TC/HDL-C). HFD_L mice exhibited significantly increased AI and CRF, indicating increased cardiovascular disease (CVD) risk (Figure 3F,G). Both GmO and GmOLb significantly ameliorated these indices, with GmOLb yielding the largest reduction, indicating superior cardioprotection. This effect may stem from the fermentation-induced enhancement of GmOLb’s fatty acid profile and fat-soluble micronutrients (MK-4 and MK-7). Vitamin K2 activates the γ-carboxylation of matrix Gla protein (MGP), a potent inhibitor of vascular calcification. Carboxylated MGP prevents the osteogenic transformation of vascular smooth muscle cells, thereby mitigating arterial calcification and stiffness, i.e., primary contributors to cardiovascular morbidity [63,64]. In addition to vascular health, vitamin K2 supports metabolic regulation. Carboxylated osteocalcin, another K2-dependent protein, enhances insulin secretion and adiponectin expression, thereby enhancing insulin sensitivity and lipid metabolism. Consistently, MK-7 supplementation in humans reduces fasting insulin and leptin levels, parallel to our observations in GmOLb-supplemented mice [65,66]. Furthermore, HFD-induced dyslipidemia contributes to leptin resistance and adiposity; however, the combination of PUFAs and vitamin K2 in GmOLb likely exerts synergistic effects by enhancing lipid metabolism, reducing inflammation, and supporting energy homeostasis.

### 3.6. Effects on Hepatic Enzyme and Bilirubin Levels in HFD-Fed Mice

**Figure 4:**
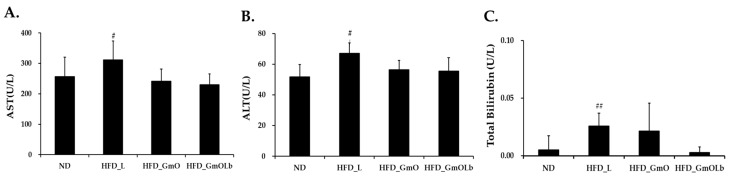
Effects of GmO and GmOLb on hepatic enzyme and bilirubin levels in HFD-fed mice. (**A**) AST, (**B**) ALT, and (**C**) total bilirubin levels after 60 days of dietary intervention. Data are expressed as the mean ± standard deviation (*n* = 8). Statistical significance: ^#^ *p* < 0.05, ^##^ *p* < 0.01 vs. ND. ND, normal diet; HFD, high-fat diet; GmO, Gwamegi oil; GmOLb, Gwamegi oil fermented with *L. brevis* KCCM13538P; AST, aspartate aminotransferase; ALT, serum alanine aminotransferase.

To assess the hepatoprotective potential of GmO and GmOLb, the serum levels of AST, ALT, and total bilirubin were measured after 60 days of treatment in HFD-fed mice (Figure 4). Mice receiving HFD supplemented with HFD_L exhibited a significant increase in AST and ALT levels than those in the ND group, indicating liver injury associated with lipid accumulation and inflammation. A slight but significant increase in total bilirubin levels was observed, indicating mild cholestatic impairment. In contrast, both HFD_GmO and fermented HFD_GmOLb attenuated the increase in AST and ALT levels compared to HFD_L, indicating reduced hepatocellular damage. Notably, the GmOLb group demonstrated a significant reduction, with enzyme levels approaching those of the ND controls. Similarly, GmOLb significantly reduced total bilirubin levels, supporting enhanced hepatic function and bile excretion. The enhanced efficacy of GmOLb may stem from fermentation-induced increases in ω-3 PUFAs (EPA and DHA) that regulate AMP-activated protein kinase and PPARα pathways to alleviate inflammation and steatosis [67]. In addition, increased menaquinones (MK-4 and MK-7) in GmOLb exert anti-fibrotic and antioxidative effects by suppressing pro-inflammatory cytokines and stabilizing mitochondrial redox balance [68,69]. These findings indicate that GmOLb enhances systemic lipid metabolism and confers robust protection against HFD-induced hepatic dysfunction. Fermentation appears to synergistically enhance the bioactivity of GmO by enriching its lipid profile and micronutrient content. In this study, we assessed how fermentation with *L. brevis* KCCM13538P (GmOLb) modifies the fatty acid and micronutrient compositions of herring and saury oils. We also assessed the therapeutic efficacy of GmOLb in diet-induced obesity and metabolic dysfunction models. Through a series of in vivo and in vitro experiments, GmOLb con sistently demonstrated potent anti-obesity, lipid-lowering, hepatoprotective, and cardiometabolic effects. These findings highlight the potential of lactic acid bacterial fermentation to enhance the physiological activity of marine oils, thereby transforming them into functional foods with significant health-promoting potential.

## 4. Discussion

The increasing global prevalence of obesity and its associated health complications, including metabolic syndrome and liver dysfunction, underscores the need for effective preventive and therapeutic strategies. In this study, we assessed the anti-obesity and hepatoprotective effects of GmO and GmOLb, produced using *L. brevis* KCCM13538P. Our findings revealed that microbial fermentation significantly enhances the bioactivity of fish oil, making GmOLb a promising candidate for treating obesity-associated metabolic disorders.

In vitro experiments using 3T3-L1 adipocytes demonstrated that GmOLb exhibited stronger anti-adipogenic effects than non-fermented GmO. GmOLb significantly suppressed lipid accumulation and intracellular TG content in differentiated adipocytes in a dose-dependent manner. These findings align with those of previous studies reporting that fermented products, particularly those involving lactic acid bacteria, inhibit adipogenesis by modulating key pathways related to lipid synthesis and storage [26,27]. The enhanced efficacy of GmOLb may be attributed to the enzymatic activity of *L. brevis*, including esterase and lipase, which can hydrolyze complex lipids and increase the release of bioavailable ω-3 PUFAs during fermentation [22]. Microbial fermentation is thus a promising strategy for enhancing the functional properties of marine-derived oils [70,71,72]. In this study, although the increases in ω-3 PUFAs (EPA and DHA) following *L. brevis* fermentation of GmO were not statistically significant, the fermented oil (GmOLb) exhibited superior physiological effects in vivo. GmOLb significantly improved serum lipid profiles, reduced visceral adiposity, and attenuated hepatic injury markers in HFD-induced obese mice. These findings suggest that fermentation may enhance the health benefits of fish oil not only through changes in fatty acid composition, but also by increasing bioavailability or generating bioactive metabolites. Further studies incorporating metabolomic profiling and dose–response analyses are warranted to elucidate the underlying mechanisms.

Our in vivo studies confirmed the superior anti-obesity effects of GmOLb. In HFD-induced obese mice, GmOLb significantly attenuated body weight gain and reduced epididymal white adipose tissue mass, a crucial indicator of visceral obesity. This aligns with studies demonstrating that ω-3-rich fish oils can mitigate diet-induced obesity by facilitating fatty acid oxidation and reducing adipogenesis [28,29]. However, the more significant effects of GmOLb indicate that fermentation amplifies these benefits. The enhanced efficacy of GmOLb can be attributed to the increased bioavailability of ω-3 PUFAs or the synergistic effects of other bioactive compounds produced during fermentation, which may affect satiety, energy expenditure, or lipid absorption [30,31].

Furthermore, GmOLb significantly enhanced serum lipid profiles in HFD-fed mice, characterized by reduced total cholesterol, TG, and LDL-C levels, and increased HDL-C levels. These changes significantly reduced the AI and CRF ratio, indicating a reduced risk of CVD. Obesity often leads to dyslipidemia owing to impaired lipid metabolism and increased synthesis of very LDL in the liver [32]. The ability of GmOLb to normalize these lipid parameters highlights its potential in ameliorating metabolic complications. The ω-3 PUFAs in fish oils are well-known for their hypolipidemic effects, primarily by suppressing hepatic TG synthesis and facilitating fatty acid oxidation [8]. Fermentation may further potentiate these effects by enhancing fatty acid absorption or producing metabolites that effectively regulate lipid metabolism. In this study, GmOLb supplementation significantly improved serum lipid profiles in HFD-fed mice, with significant reductions in total cholesterol, TG, and LDL-C, and a significant increase in HDL-C. These changes resulted in lower AI and CRF values, indicating enhanced cardiovascular protection.

A significant finding was the potent hepatoprotective effect of GmOLb. HFD-induced obesity often causes NAFLD, characterized by hepatic steatosis, inflammation, and potential progression to more severe liver pathologies [33]. Our results demonstrated that GmOLb significantly reduced the increased serum levels of ALT, AST, and total bilirubin and ameliorated hepatic steatosis, as confirmed by histopathological analysis. These findings indicate that GmOLb effectively protects the liver from HFD-induced damage. The hepatoprotective mechanism of ω-3 PUFAs involves reducing hepatic lipid accumulation, oxidative stress, and inflammatory responses within the liver [9,34]. The enhanced efficacy of GmOLb may stem from the increased bioavailability of ω-3 PUFAs because of fermentation or the production of specific anti-inflammatory or antioxidant compounds by *L. brevis*. For example, certain bacterial metabolites modulate the gut microbiota, thereby influencing liver health by affecting gut barrier integrity and reducing the translocation of bacterial endotoxins to the liver [35]. Despite the observed improvements in adiposity and lipid profiles, this study has some limitations. The short duration, single-dose design, and absence of dose optimization limit conclusions regarding long-term efficacy and safety. In addition, the inherent variability in individual responses and the use of a murine model restrict the direct applicability of the findings to human physiology. Future studies should incorporate extended intervention periods, dose–response analyses, and clinical validation to substantiate the therapeutic potential of GmOLb.

Our study demonstrates the promising effects of GmOLb; however, certain additional limitations warrant consideration. First, the mechanisms by which *L. brevis* fermentation enhances the bioactivity of GmO remain unclear. In this study, a small amount of yeast extract (0.1%) was added to support microbial growth and metabolic activity during lipid-based fermentation. Fish oil served as the primary substrate; however, this auxiliary nitrogen source may have influenced microbial metabolism and fermentation outcomes. In addition to improving lipid composition, *L. brevis* fermentation may also generate short-chain fatty acids (SCFAs), including acetate, propionate, and butyrate, through partial metabolism of available substrates. SCFAs are known to exert anti-obesity effects by modulating lipid metabolism, appetite regulation, and inflammation. While SCFA levels were not measured in this study, their potential contribution to the observed metabolic benefits cannot be excluded. Future studies should investigate the effects of different fermentation substrates, monitor SCFA production, and perform targeted metabolomic analyses to identify key fermentation-derived bioactives and elucidate their molecular mechanisms of action. Second, although the in vivo model provided valuable insights, long-term studies are needed to evaluate the sustained efficacy and safety of GmOLb. Finally, investigating the effects of GmOLb on gut microbiota composition may offer a more comprehensive understanding of its metabolic impact. While ω-3 PUFAs and fat-soluble vitamins likely play a central role, additional bioactive compounds—such as SCFAs, lipid mediators (e.g., resolvins), bioactive peptides, conjugated linoleic acids, and medium-chain triglycerides—may also contribute to the observed physiological benefits. These effects may be mediated by fermentation-derived SCFAs and modified lipids, both of which have been shown to regulate energy metabolism and inflammatory responses [70,71]. In addition to ω-3 PUFAs, fermented marine oils may be enriched with micronutrients such as vitamin K2 (menaquinones), which are known to exert beneficial effects on lipid metabolism and hepatic function. Menaquinone-4 (MK-4) and menaquinone-7 (MK-7), the bioactive forms of vitamin K2, have been shown to modulate insulin signaling, suppress hepatic lipogenesis, and reduce inflammation, thereby contributing to improved lipid profiles and reduced hepatic steatosis [14,15,16]. Microbial fermentation, particularly with *L. brevis*, may enhance the conversion of vitamin K1 or endogenous precursors into MK-4 and MK-7, thereby increasing the bioavailability of these fat-soluble micronutrients. Vitamin K levels were not quantified in this study; however, the potential enrichment of menaquinones during fermentation represents a promising mechanistic link that may explain the improved metabolic outcomes observed with GmOLb. Future studies should include the targeted quantification of MK vitamins, and investigate their synergistic interactions with ω-3 PUFAs and other fermentation-derived bioactives in regulating lipid and liver metabolism. While polyphenols and terpenes are not typically associated with fish-derived oils, these fermentation-related metabolites warrant further investigation.

## 5. Conclusions

This study demonstrates that fermenting marine oils—specifically herring and saury—with *L. brevis* KCCM13538P (GmOLb) significantly enhances their nutritional and functional profiles. Fermentation enhanced the fatty acid composition (notably increasing ω-3 PUFAs) and micronutrient levels (MK-4 and MK-7). In both in vivo and in vitro models of diet-induced obesity and metabolic dysfunction, GmOLb exhibited strong anti-obesity, lipid-lowering, hepatoprotective, and cardiometabolic effects. These consistent and multifaceted therapeutic effects support the conclusion that lactic acid fermentation significantly enhances the bioactivity of marine oils. Therefore, GmOLb is a promising candidate as a functional food supplement with significant potential for preventing or mitigating obesity-associated metabolic disorders and liver dysfunction. Future research on long-term safety, human clinical efficacy, and mechanistic pathways will be essential to fully use its nutraceutical potential.

## Figures and Tables

**Table 1:** Fatty acid composition of GmO and GmOLb.

Fatty Acid (g/100 g)	Lipid Number	GmO	GmOLb	Type
SFA (Saturated Fatty Acid)				
Myristic acid	C14:0	4.28 ± 0.21	4.33 ± 0.22	
Pentadecanoic acid	C15:0	0.37 ± 0.02	0.37 ± 0.03	
Palmitic acid	C16:0	13.03 ± 0.65	12.32 ± 0.62	
Heptadecanoic acid	C17:0	0.31 ± 0.02	0.35 ± 0.02	
Stearic acid	C18:0	2.74 ± 0.14	2.6 ± 0.13	
Arachidic acid	C20:0	0.23 ± 0.01	0.21 ± 0.01	
Eicosanoic acid	C20:0	3.33 ± 0.17	3.37 ± 0.17	
Heneicosylic acid	C21:0	0.07 ± 0.01	0.07 ± 0.01	
Behenic acid	C22:0	0.08 ± 0.03	0.08 ± 0.03	
Total SFAs (g/100 g)		24.44 ± 0.72	23.7 ± 0.69	
USFA (Unsaturated Fatty Acid)				
Myristoleic acid	C14:2	0.07 ± 0.01	0.0 7± 0.02	MUFA (ω-5)
Palmitoleic acid	C16:1	6.71 ± 0.34	6.92 ± 0.35	MUFA (ω-7)
Oleic acid	C18:1	19.2 ± 0.96	20.03 ± 1.02	MUFA (ω-9)
Linoleic acid	C18:2	2.74 ± 0.14	2.79 ± 0.14	PUFA (ω-6)
α-Linolenic acid	C18:3	1.33 ± 0.07	1.39 ± 0.07	PUFA (ω-3)
γ-Linolenic acid	C18:3	0.01 ± 0.00	0.15 ± 0.01	PUFA (ω-6)
Elaidic acid	C18:1	0.16 ± 0.01	0.74 ± 0.04	Trans-MUFA (ω-9)
Linolelaidic acid	C18:2	0	0.15 ± 0.02	Trans-PUFA (ω-6)
Eicosadienoic acid	C20:2n-6	0.28 ± 0.01	0.16 ± 0.21	PUFA (ω-6)
Eicosatetraenoic acid	C20:4	0.12 ± 0.01	0.09 ± 0.02	PUFA (ω-3)
Eicosatrienoic acid	C20:3n-3	0.15 ± 0.01	0.13 ± 0.01	PUFA (ω-3)
Arachidonic acid	C20:4	0.61 ± 0.03	0.64 ± 0.02	PUFA (ω-6)
Eicosapentaenoic acid (EPA)	C20:5	7.56 ± 0.38	7.66 ± 0.38	PUFA (ω-3)
Erucic acid	C22:1	0.54 ± 0.03	0.48 ± 0.02	MUFA (ω-9)
Docosapentaenoic acid	C22:5	0.59 ± 0.03	0.62 ± 0.03	PUFA (ω-3)
Docosahexaenoic acid (DHA)	C22:6	8.92 ± 0.45	9.13 ± 0.46	PUFA (ω-3)
Nervonic acid	C24:1	0.44 ± 0.02	0.51 ± 0.03	MUFA (ω-9)
Total USFAs (g/100 g)		49.43 ± 1.19	51.66 ± 1.26	

GmO: Gwamegi oil, GmOLb: fermented Gwamegi oil, and MUFA: monounsaturated fatty acid. Values are expressed as the mean ± standard deviation (estimated). Statistical comparisons were not performed. Data represent the sum of quantified fatty acids detected using gas chromatography; trace-level and unidentified fatty acids are not included.

**Table 2:** Contents of fat-soluble vitamins in GmO and GmOLb.

Vitamin	Abbreviation	Unit (/100 g)	GmO	GmOLb
Vitamin A	Vit A	μg RE	6999.41 ± 34.99	7579.27 ± 37.89
Vitamin D	Vit D	μg	56.91 ± 0.28	67.95 ± 0.34
Vitamin E	Vit E	μg α-TE	1070 ± 6.85	4780 ± 23.91
Vitamin K2	MK-4	μg	42.19 ± 3.26	64.13 ± 3.07
	MK-7	μg	19.28 ± 1.48	46.56 ± 1.82

GmO: Gwamegi oil and GmOLb: fermented Gwamegi oil. Values are expressed as the mean ± standard deviation per 100 g of sample. The experiments were conducted in triplicate. Vitamin A is expressed as retinol equivalents (RE); vitamin D as micrograms (μg); vitamin E as alpha-tocopherol equivalents (α-TE); and vitamin K2 as menaquinone-4 (MK-4) and menaquinone-7 (MK-7), both in μg.

## Data Availability

The original contributions presented in this study are included in the article. Further inquiries can be directed to the corresponding authors.
